# Efficacy and Safety of Q10 Ubiquinol With Vitamins B and E in Neurodevelopmental Disorders: A Retrospective Chart Review

**DOI:** 10.3389/fpsyt.2022.829516

**Published:** 2022-03-03

**Authors:** Francesca Cucinotta, Arianna Ricciardello, Laura Turriziani, Arianna Mancini, Roberto Keller, Roberto Sacco, Antonio M. Persico

**Affiliations:** ^1^Interdepartmental Program “Autism 0-90”, “G. Martino” University Hospital, Messina, Italy; ^2^IRCCS Centro Neurolesi “Bonino-Pulejo”, Messina, Italy; ^3^Villa Miralago, Cuasso al Monte, Italy; ^4^Mental Health Department, Adult Autism Centre, Rete Ospedaliera Territorio Nord-Ovest, Azienda Sanitaria Locale Città di Torino, Turin, Italy; ^5^Service for Neurodevelopmental Disorders, University “Campus Bio-Medico”, Rome, Italy; ^6^Child and Adolescent Neuropsychiatry Program, Modena University Hospital and Department of Biomedical, Metabolic and Neural Sciences, University of Modena and Reggio Emilia, Modena, Italy

**Keywords:** Autism Spectrum Disorder, coenzyme Q10, Intellectual Disability, neurodevelopmental disorders, Phelan-McDermid syndrome, Ubiquinol-10, vitamin B, vitamin E

## Abstract

Increased oxidative stress and defective mitochondrial functioning are shared features among many brain disorders. The aim of this study was to verify retrospectively the clinical efficacy and safety of a metabolic support therapy with Q10 ubiquinol, vitamin E and complex-B vitamins in various neurodevelopmental disorders. This retrospective chart review study included 59 patients (mean age 10.1 ± 1.2 y.o., range 2.5–39 years; M:F = 2.47:1), diagnosed with Autism Spectrum Disorder (*n* = 17), Autism Spectrum Disorder with co-morbid Intellectual Disability (*n* = 19), Intellectual Disability or Global Developmental Delay (*n* = 15), Attention-Deficit/Hyperactivity Disorder (*n* = 3) and Intellectual Disability in Phelan-McDermid syndrome due to chr. 22q13.33 deletion (*n* = 5). After a minimum of 3 months of therapy, a positive outcome was recorded in 45/59 (76.27%) patients, with Clinical Global Impression—Improvement scores ranging between 1 (“very much improved”) and 3 (“minimally improved”). The most widespread improvements were recorded in cognition (*n* = 26, 44.1%), adaptative functioning (*n* = 26, 44.1%) and social motivation (*n* = 19, 32.2%). Improvement rates differed by diagnosis, being observed most consistently in Phelan-McDermid Syndrome (5/5, 100%), followed by Intellectual Disability/Global Developmental Delay (13/15, 86.7%), Autism Spectrum Disorder with co-morbid Intellectual Disability (15/19, 78.9%), Autism Spectrum Disorder (11/17, 64.7%) and ADHD (1/3, 33.3%). No significant adverse event or side effect leading to treatment discontinuation were recorded. Mild side effects were reported in 18 (30.5%) patients, with the most frequent being increased hyperactivity (9/59, 15.3%). This retrospective chart review suggests that metabolic support therapy with Q10 ubiquinol, vitamin E and complex-B vitamins is well tolerated and produces some improvement in the majority of patients with neurodevelopmental disorders, especially in the presence of intellectual disability. Randomized controlled trials for each single neurodevelopmental disorder are now warranted to conclusively demonstrate the efficacy of these mitochondrial bioenergetic and antioxidant agents and to estimate their therapeutic effect size.

## Introduction

Converging data from animal models and human research indicate that oxidative stress likely represents a shared feature present in many brain disorders and more specifically in neurodevelopmental disorders (NDDs), including Autism Spectrum Disorder (ASD), Attention-Deficit/Hyperactivity Disorder (ADHD), and Intellectual Disability (ID) (1–3). Although enhanced oxidative stress is usually the consequence and not the primary cause of NDDs (4), reduced ATP production and oxidative damage can seemingly contribute an additional burden to the dysfunction directly produced by the genetic and/or epigenetic defects directly responsible for each disorder. Within this framework, supporting mitochondrial function and reducing oxidative stress, while not correcting the primary mechanism responsible for abnormal neurodevelopment, may partially improve behavioral symptoms in many patients fulfilling DSM-5 diagnostic criteria for an heterogeneous array of neurodevelopmental conditions (5).

ASD represents a paradigmatic example of the “ying-yang” role of oxidative stress and mitochondrial dysfunction, at the same time “consequence” and “cause” of abnormal neurodevelopment, as best investigated in ASD compared to other NDDs (4). ASD is a heterogeneous neurodevelopmental condition characterized by impairment in language, communication and social skills, as well as by repetitive and stereotypic behaviors, restricted interests and abnormal sensory-processing, with onset in early childhood (5). ASD etiology includes a wide range of medical, genetic, epigenetic/environmental, immunological, biochemical conditions or factors (6), all converging on a limited number of functional pathways, clustered around three major themes: neurodevelopment, chromatin organization, and immune function (7–9). Pharmacological treatments are prescribed to correct comorbid symptoms in ASD, like sleep disorders, aggressiveness and irritability, hyperactivity and attention deficit, while no effective pharmacological therapy is currently available for the core symptoms of ASD (10). These symptoms respond to interventions based on different cognitive-behavioral paradigms, which are indeed effective but also long-lasting, expensive, and time consuming. Furthermore, their efficacy displays great interindividual variability depending not only on treatment approach, therapist experience, and therapeutic setting, but also on the genetic background of the patient (11). In this context, any pharmacological treatment able to at least partly ameliorate core ASD symptoms, could conceivably increase the efficacy of behavioral interventions and promote better adaptive functioning, ultimately improving the quality of life of patients and families.

Many studies have consistently described enhanced oxidative stress and impaired mitochondrial metabolism in ASD both peripherally and in post-mortem brains (1, 4, 12–18). Oxidative damage has been detected in neocortical regions involved in speech processing, social interaction, memory, sensory and motor coordination, especially the superior temporal gyrus, frontal cortex and cerebellum, but not in the parietal and occipital cortices (14–18). Importantly, redox abnormalities have been detected also in young autistic children and are not correlated with age: enhanced oxidative stress and mitochondrial dysfunction thus seemingly represent an ASD-related “state-dependent” characteristic produced by abnormal neurodevelopment and are present in a consistent number of autistic individuals regardless of their age and of their specific underlying pathogenetic underpinnings. Meanwhile, although oxidative stress usually represents the consequence and not the primary cause of ASD (4, 13), reduced ATP production and oxidative damage can contribute an additional burden to the dysfunction directly produced by ASD-causing genetic or epigenetic defects, resulting in greater symptom severity (12). Sustaining mitochondrial function while controlling redox imbalance may thus represent a viable “indirect” therapeutic approach, potentially able to ameliorate behavioral and neuropsychological deficits in many individuals with ASD and possibly with other NDDs (19–21).

Coenzyme Q10 (CoQ10, ubiquinone) is a lipid soluble compound present in the majority of living cells. In humans, once administered per os it is absorbed in the gut and reduced in the liver to its active form, Q10-ubiquinol, which acts as a diffusible electron carrier in the mitochondrial respiratory chain, and as an antioxidant removing free radicals (22–24). CoQ10 has been proven effective in diverse experimental disease paradigms of oxidative stress (25–27). Therefore, by increasing energy production and antioxidant capacity, CoQ10 is predicted to limit the damage generated by the neuroinflammationm microglial activation, and excitotoxicity well documented in ASD brains, ultimately leading to excessive neuritic pruning and/or cell apoptosis (28, 29). Improvements in ASD using nutritional supplements which can support mitochondrial function and reduce oxidative stress, also including CoQ10, have been reported (30–32). However, these studies are limited in sample size (30), or have used Q10-ubiquinone which is less readily absorbed by the gut and requires biotransformation into ubiquinol (31, 32), or have administered a cocktail of many different compounds, partly sharing antioxidant properties but also exerting many other actions which escape experimental control (31). In the present study, we report the results of a systematic chart review involving all patients with neurodevelopmental disorders (NDDs), treated in our clinical Units for a minimum of 3 months with a “metabolic support therapy” (MST) consisting in a small and targeted set of compounds, namely Q10 ubiquinol, vitamin E and complex-B vitamins at fixed daily doses. Our retrospective study provides encouraging results, supporting the efficacy of this treatment approach in the majority of patients, albeit with interindividual heterogeneity and to a different extent in different NDDs.

## Materials and Methods

We performed a retrospective chart review of all cases with any NDD consecutively admitted as outpatients or inpatients to the Interdepartmental Program “Autism 0-90” of the “G. Martino” University Hospital in Messina (Italy) and to the Adult Autism Centre of the ASL “Città di Torino” in Turin (Italy) between June 1, 2016 and December 31, 2020, and treated with MST for a minimum of 3 months. MST encompasses Q10 ubiquinol (50–100 mg b.i.d.), vitamin E (30–60 mg b.i.d.) and B-group vitamins, including nicotinamide, dexpanthenol, riboflavin-5'-sodium phosphate, inositol, pyridoxine hydrochloride, and cyanocobalamin (Table 1). Two different dose levels have been prescribed in our clinical practice for each compound based on body weight, with the lower dose prescribed to children ≤ 20 kg and the higher dose to patients weighing > 20 kg (Table 1). Vitamins E and group-B were dosed according to the maximum daily intake of vitamins and minerals allowed in food supplements by the Italian Ministry of Health (33). Two Child Neuropsychiatrists (LT, AM) not involved in the clinical management of these patients reviewed all medical charts from both sites and independently rated patients, using the Clinical Global Impression of Severity (CGI-S) scale, applied to information collected at patient intake, as well as the Clinical Global Impression of Improvement (CGI-I) scale after a minimum of 3 months of MST treatment (34). The data retrieved from chart review included patient sex, age, DSM-5 diagnosis, main symptoms, response to treatment and adverse effects, as recorded by the clinician following each patient. Parental reports and observations by therapists, blind to the beginning of MST, were recorded during visits and were considered in determining CGI-I scores, as they provide valuable information especially in reference to adaptative functioning and social motivation. All diagnoses were made by an experienced Child and Adolescent or Adult Psychiatrist, according to the Diagnostic and Statistical Manual of Mental Disorders, 5th edition (DSM-5) (5) based on direct observation, a semi-structured play session or interview depending on patient age and cognitive level, and parental report. The clinical diagnosis was further confirmed using the Autism Diagnostic Observation Schedule – 2nd ed (ADOS-2) and the Autism Diagnostic Interview-Revised (ADI-R) for ASD, the Griffiths Mental Development Scale (GMDS) for GDD, a cognitive test (Leiter-R, WPPSI-III, WISC-IV, WAIS-IV) for ID, the Conners Parent Rating Scale (CPRS) also in Teacher version (TRF) and the Batteria Italiana per l'ADHD (BIA) for ADHD. A standard panel of psychodiagnostic tests was also applied to assess executive functions, learning abilities, adaptive level, psychoeducational profile and so on, depending on DSM-5 diagnosis and clinical characteristics of each patient. Written informed consent to the use for scientific and publication purposes of data derived from clinical outpatient and inpatient work-up was collected from both parents/legal guardian of all patients. A separate written consent regarding the publication of drawings depicted in **Figure 3B** was provided by both parents.

**Table 1:** Composition of the “metabolic support therapy”.

**Compounds**	**Daily dose by patient weight**	
	**≤20 kg**	**>20 kg**
Q10 ubiquinol	50 mg	100 mg
Vitamin E	30 mg	60 mg
Vitamin B2 (riboflavin HCl)	0.45 mg	0.90 mg
Vitamin B3 (Niacin)	5.5 mg	11 mg
Vitamin B5 (Pantothenic acid)	3 mg	6 mg
Vitamin B6 (Pyridoxine)	0.45 mg	0.90 mg
Vitamin B12 (Cyanocobalamine)	0.70 mcg	1.40 mcg
Inositol	5 mg	10 mg

## Results

**Figure 1:**
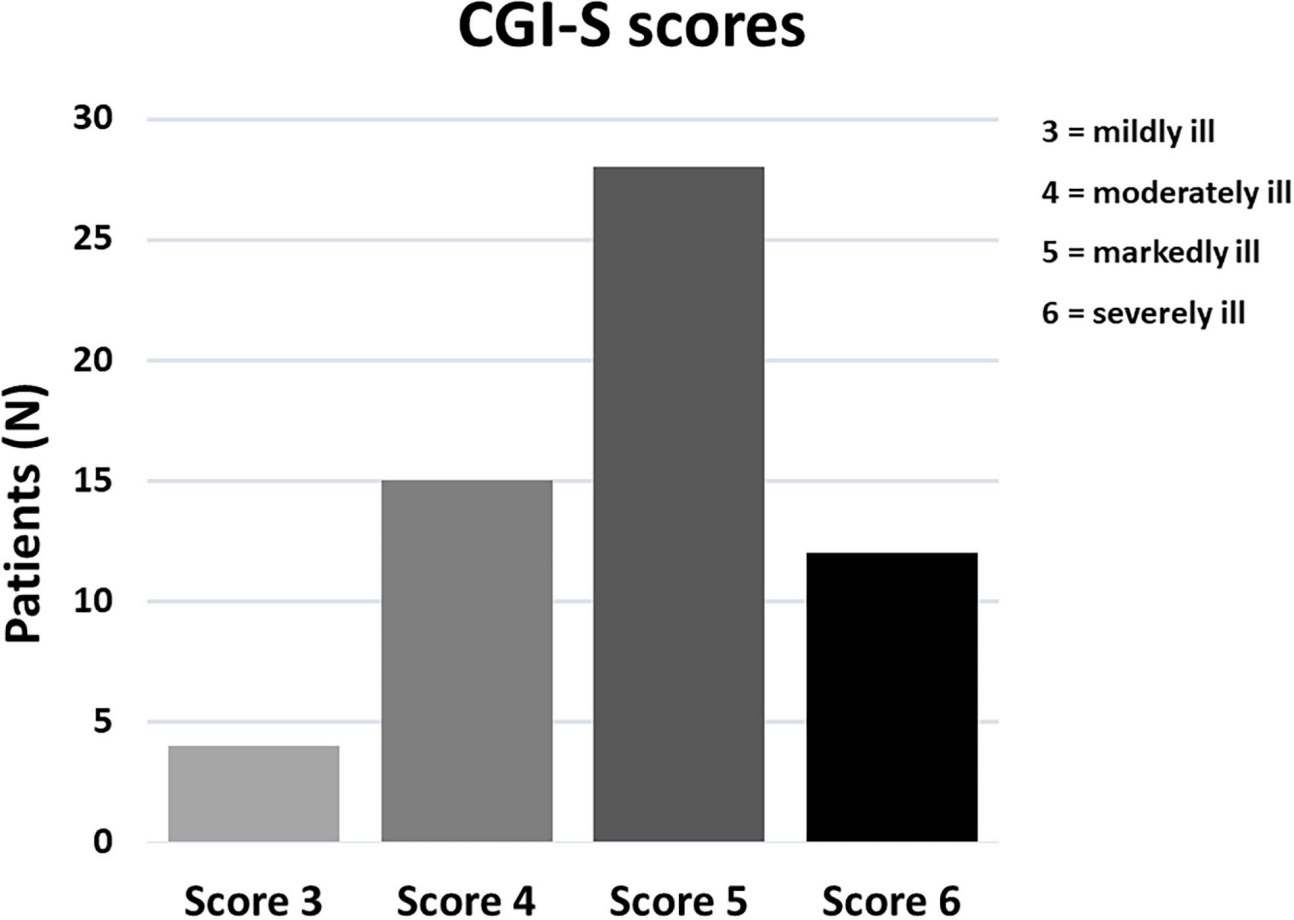
Distribution of CGI-S severity scores in our sample of 59 patients with neurodevelopmental disorders.

A total of 59 patients with NDDs satisfied the study selection criteria and their charts were reviewed. Among them, 49 children or adolescents and 6 young adults were recruited at the Messina site, while 4 young adults were recruited at the Turin site. The demographic and clinical characteristics of the sample are summarized in Table 2. Age ranges between 2.5 and 39 y.o. (mean 10.09 y.o.) and the M:F ratio is 2.47. This sex ratio is in line with an excess of males being affected by neurodevelopmental disorders within all DSM-5 diagnostic categories, ranging from approximately M:F = 1.5:1 in ID to 4:1 in ASD (6). Patients had a variety of primary NDD diagnoses, encompassing severe (“level 3”) ASD with co-morbid ID or Global Developmental Delay (GDD) (*n* = 19, 32.2%), moderate or mild (“levels 1–2”) ASD (*n* = 17, 28.8%), ID or GDD (*n* = 15, 25.4%), ADHD (*n* = 3, 5.1%) and 5 (8.5%) patients with Phelan-McDermid Syndrome (PMS), all characterized by the presence of ID, severe Language Developmental Disorder especially for expressive language, Motor Coordination Disorder and autistic traits up to a full ASD diagnosis. The majority of patients were severely affected prior to treatment, with 40/59 (67.8%) rated as “markedly ill” or “severely ill” using the CGI-S (Figure 1). Psychopharmacological treatments for the management of behavioral symptoms were being taken by 18 (30.5%) patients at baseline, with the most commonly prescribed medications being atypical antipsychotics which were maintained constant during at least 3 months of MST.

**Table 2:** Demographic and clinical characteristics of the sample (*N* = 59).

Age in yrs (mean ± S.E.M. and range)	10.09 ± 1.20 (2.5–39)	
Gender	Male	42
	Female	17
M:F ratio		2.47:1
DSM-5 diagnosis		
• ASD with Intellectual Disability or Global Developmental Delay		19 (32.2%)
• ASD, with or without ADHD		17 (28.8%)
• Intellectual Disability or Global		15 (25.4%)
Developmental Delay		
• Intellectual Disability in Phelan-McDermid Syndrome		5 (8.5%)
• ADHD		3 (5.1%)
ASD severity level		
1 – Mild		8 (22.2%)
2 – Moderate		17 (47.2%)
3 - Severe		11 (30.6%)
Intellectual disability level		
• Mild		21 (53.8%)
• Moderate		4 (10.3%)
• Severe/profound		14 (35.9%)
ADHD subtype		
• Mixed		6 (60.0%)
• Inattentive		4 (40.0%)

**Figure 2:**
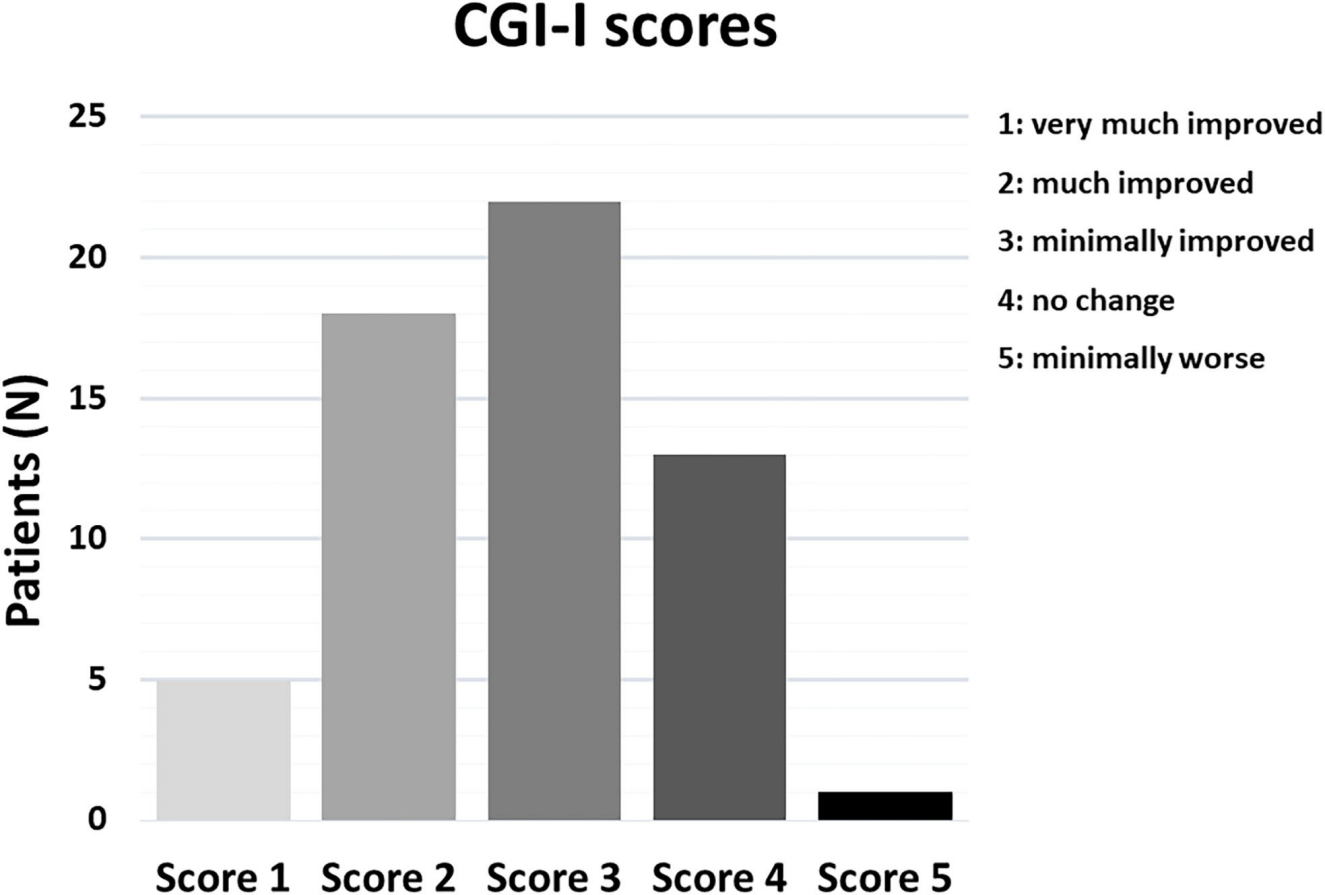
Distribution of CGI-I clinical improvement scores.

**Figure 3:**
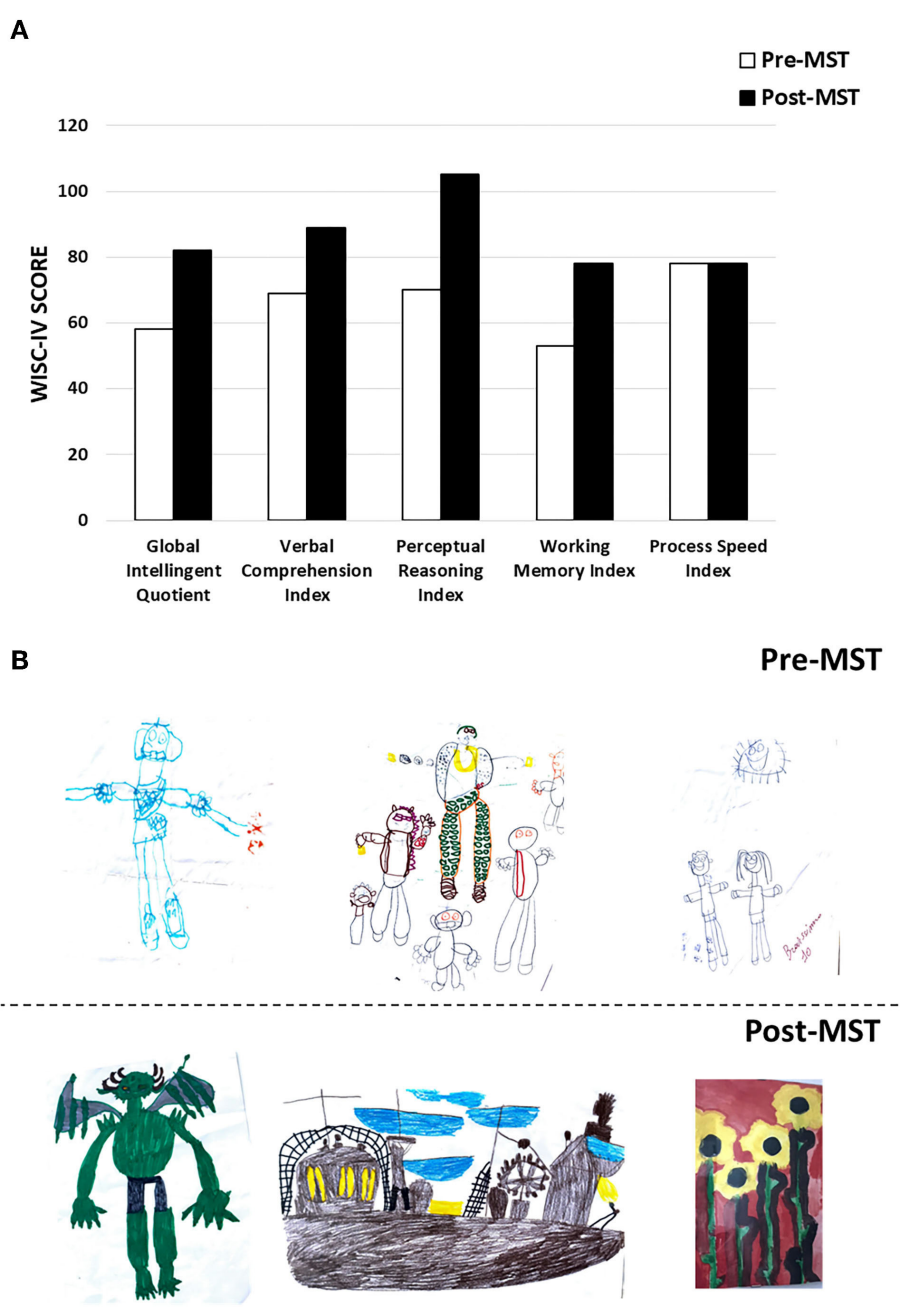
Two “very much improved” patients: **(A)** cognitive profile of a 13 year-old boy with ID before and after 6 months of MST, assessed using the WISC-IV; **(B)** drawings by a 12 year-old boy with mild ASD before and after 3 months of MST.

**Figure 4:**
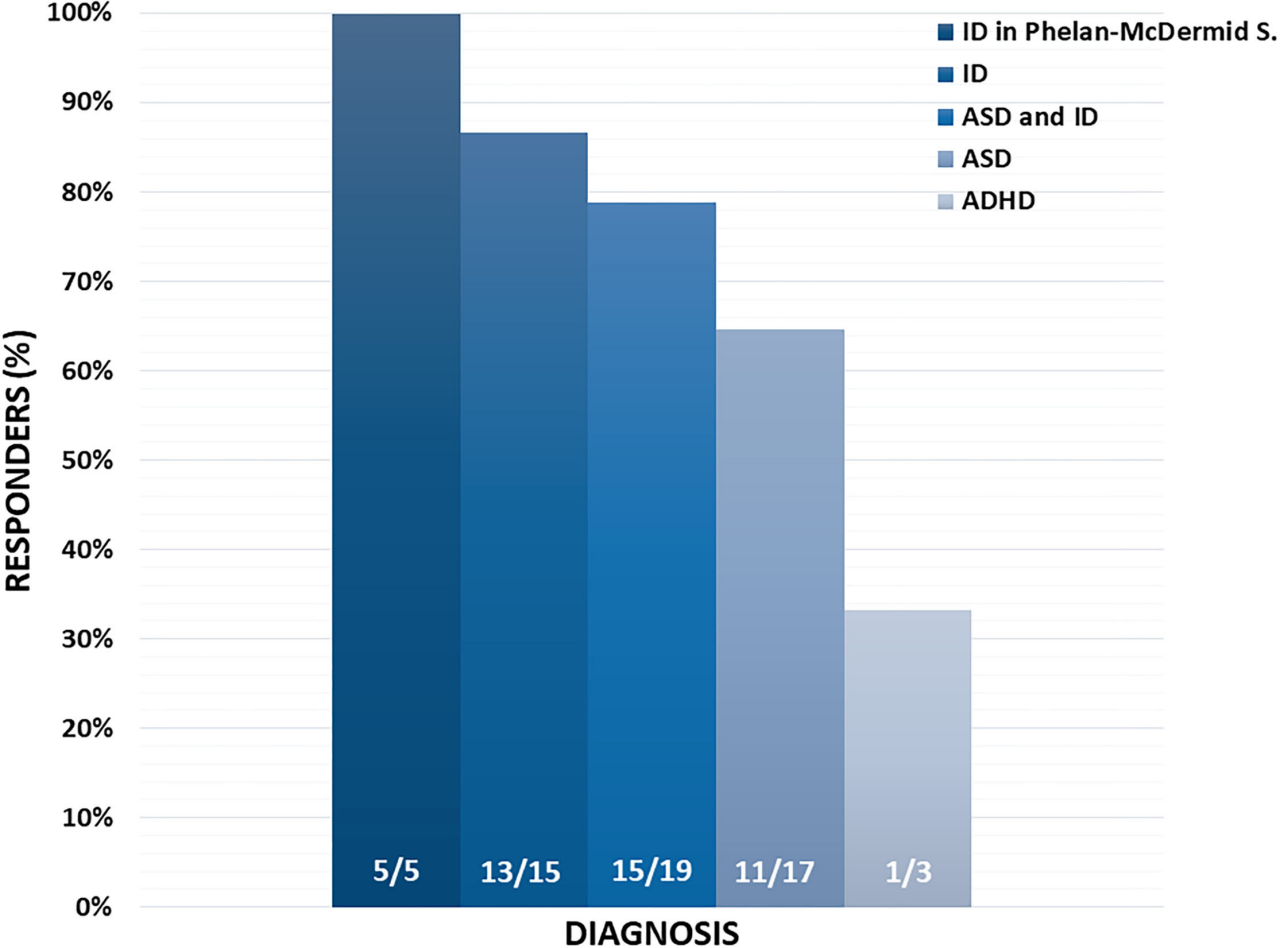
Percentage and number of responders by diagnostic category.

Based on clinical chart reviews, 45/59 (76.27%) patients were rated as “responders” to MST according to CGI-I scores (Figure 2). The most consistent improvements were recorded in the areas of cognition and adaptive functioning, with parents and clinicians reporting particularly improved perception of the surrounding environment and greater responsiveness to environmental stimuli in 26/59 (44%) of the patients (Table 3). Also social interactions, motor coordination, attention, and language skills improved in a sizable 22–32% of the sample (Table 3). Lower rates of response were reported for emotional dysregulation, hyperactivity, anxiety, and stereotypic behaviors, at ~3–9% of the sample (Table 3). Two paradigmatic examples of “very much improved” cases are presented in Figure 3: (A) a 13 year-old boy diagnosed with ID who after 6 months of MST gained over 20 IQ points at the WISC-IV (35) and normalized visual and auditory attention scores at the NEPSY-II (36); (B) improved drawing skills by a 12 year-old boy with mild ASD after 3 months of MST. A greater emotional use of colors is also evident, pointing toward possible antidepressant effects which we have observed in some children and that will deserve targeted studies. Response rates may differ depending on diagnosis, with higher percentages of responders associated with the presence of ID as compared to ASD or ADHD (Figure 4), although this trend is not significant in the total sample (Chi sq. exact *P* = 0.127, after 10,000 iterations), nor for each single functional domain after controlling for multiple testing (Table 3, nominal exact *P*-values <0.05 only for motor coordination and emotional self-regulation).

**Table 3:** Therapeutic effects recorded following metabolic support therapy in the entire sample and by DSM-5 diagnosis.

**Improved function/decreased symptom**	***N* (%) of patients**	**DSM-5 diagnosis**				
		**ASD + ID/GDD**	**ASD ±ADHD**	**ID/GDD**	**ID in PMS**	**ADHD**
Cognition	26 (44.1%)	10 (52.6%)	5 (29.4%)	9 (60.0%)	1 (20.0%)	1 (33.3%)
Adaptive functioning, responsiveness to environmental stimuli.	26 (44.1%)	9 (47.4%)	5 (29.4%)	8 (53.3%)	3 (60.0%)	1 (33.3%)
Social interaction and motivation	19 (32.2%)	5 (26.3%)	3 (17.6%)	6 (40.0%)	4 (80.0%)	1 (33.3%)
Motor coordination	15 (25.4%)	5 (26.3%)	4 (23.5%)	2 (13.3)	4 (80.0%)	0 (0.0%)
Selective and sustained attention	12 (20.3%)	3 (15.8%)	4 (23.5%)	3 (20.0%)	2 (40.0%)	0 (0.0%)
Language and communication skills	13 (22.0%)	7 (36.8%)	1 (5.9%)	4 (26.7%)	1 (20.0%)	0 (0.0%)
Emotional self-regulation	5 (8.5%)	5 (26.3%)	0 (0.0%)	0 (0.0%)	0 (0.0%)	0 (0.0%)
Hyperactivity	4 (6.8%)	3 (15.8%)	1 (5.9%)	0 (0.0%)	0 (0.0%)	0 (0.0%)
Anxiety	3 (5.1%)	2 (10.5%)	0 (0.0%)	0 (0.0%)	1 (20.0%)	0 (0.0%)
Stereotypic behaviors	2 (3.4%)	2 (10.5%)	0 (0.0%)	0 (0.0%)	0 (0.0%)	0 (0.0%)
**Total** ***N***	**59 (100%)**	**19 (100%)**	**17 (100%)**	**15 (100%)**	**5 (100%)**	**3 (100%)**

*ASD, Autism Spectrum Disorder; ID, Intellectual Disability; GDD, Global Developmental Delay; PMS, Phelan-McDermid Syndrome; ADHD, Attention Deficit/Hyperactivity Disorder*.

Side effects occurred in 18/59 (30.5%) patients and are listed in Table 4. No significant adverse event or severe side effect was recorded. Side effects were always mild, never lead to treatment discontinuation and could be easily managed by parents or clinicians. These included mainly mild hyperactivity, which was usually transient, and early insomnia, which could be avoided by administering MST in mid-afternoon rather than after dinner. Only one patient (1.7%) had a “minimally worse” score at the CGI-I, and also in this case side effects were not clinically meaningful and could be easily managed.

**Table 4:** Side effects recorded during metabolic support therapy.

**Side effect**	***N* (%) of patients**
Hyperactivity	9 (15.3%)
Early insomnia	6 (10.2%)
Irritability, self-aggressiveness	4 (6.8%)
Increased stereotypic behaviors	4 (6.8%)
Oppositional behavior	3 (5.1%)
Increased appetite	2 (3.4%)

## Discussion

The present study provides retrospective evidence of efficacy and tolerability for a “metabolic support therapy” encompassing Q10-ubiquinol, Vitamin E and complex-B vitamins in patients with different neurodevelopmental disorders. In terms of efficacy, MST was associated with clinical improvement in 45/59 (76.27%) patients (Figure 2, CGI-I cat. 1-3). Medical records documented clinical improvement always through direct observation by the neuropsychiatrist and through parental report, but also as frequently reported to parents by therapists, who were unaware of the beginning of MST. Improvement was most frequently recorded in the domains of cognition, adaptive functioning, responsiveness to environmental stimuli, social interaction and motivation, motor coordination, selective and sustained attention, language and communication skills (Table 3). A sizable number of patients (23/59, 39.0%) retains “responder” status if the threshold of clinical response is raised to “much improved” and “very much improved” CGI-I scores (cat. 2 and 1, respectively, in Figure 2). Side effects were mild and always manageable. The very limited number of compounds administered in this study, as compared to some previous studies (31), allows to define more clearly the experimental question and more specifically the putative mechanisms underlying the clinical improvement observed in this sample.

Enhanced oxidative stress and consequent mitochondrial dysfunction have been described in many different brain disorders (37, 38). These include neurodevelopmental disorders, like ASD (1), ADHD (2), and ID (3); genetic disorders, like Rett (39, 40), Fragile X (41), and Down syndromes (42, 43); neurological disorders, like Alzheimer's and Parkinson's disease (44); and psychiatric disorders, like schizophrenia, depression and bipolar disorder (37, 45). A variety of etiological genetic variants and environmental agents can seemingly trigger pathogenetic mechanisms which, in addition to producing disease-specific damage, ultimately converge upon this final common pathway. Oxidative damage can occur due to either the direct effects exerted by an etiological agent on neural cells or to the indirect consequences of abnormal neurochemistry and/or dysfunctional neural activity patterns. In post-mortem brains and in muscle biopsies of children with ASD, for example, oxidative stress has been conclusively shown to produce mitochondrial dysfunction, by decreasing levels of electron transport chain complexes and impairing their function, significantly decreasing ATP synthesis (16, 46, 47). In addition to mitochondrial dysfunction and abnormal energy metabolism, other mechanisms potentially linking oxidative stress to neuronal dysfunction include membrane lipid abnormalities, neuroinflammation, and increased excitotoxicity (1–4). Preventing, correcting or at least minimizing the mitochondrial dysfunction produced by oxidative damage in brain disorders, while not directly correcting the primary mechanism responsible for each pathology, may nonetheless improve the clinical conditions, acting as an indirect therapeutic approach.

Coenzyme Q10 is a lipid soluble compound synthesized by our organism and also introduced in small amounts through the diet. It acts as an electron acceptor donor, by oscillating between its oxidized (ubiquinone) and reduced (ubiquinol) forms (22–24). This redox activity sustains mitochondrial homeostasis and prevents free radical generation. Therefore, CoQ_10_ is recognized as an intracellular antioxidant that protects membrane phospholipids, mitochondrial membrane proteins, and low-density lipoproteins from the oxidative damage produced by free radicals (22–24). Furthermore, intra-mitochondrial concentrations of CoQ_10_ largely influence the efficiency of the mitochondrial respiratory chain. For this reason, exogenous CoQ10 is routinely prescribed to children with mitochondrial myopathies to ameliorate their bioenergetic impairment (48), and has been proposed for many neurological and psychiatric conditions potentially benefitting from support of mitochondrial function, such as Alzheimer's Disease, Parkinson's Disease, Huntington's Disease, and migraine (49). Importantly, a genome-wide association analysis searching for loci associated with CoQ10 serum levels identified *COLEC12* and *NRXN-1*, which have also been associated with neuropsychiatric disorders, including Alzheimer's Disease, ASD and Schizophrenia (50).

MST is predicted to ameliorate clinical symptoms by improving brain mitochondrial function and ATP production. The focused co-administration of Q10-ubiquinol, the reduced form of CoQ10 most readily absorbed by the gut, with only two known antioxidants, vitamin E and a multivitamin B complex, is designed to synergistically boost the increase in energy production and cell protection viewed as deriving primarily from Q10 ubiquinol administration. In accordance with previous studies of ASD (30–32) and other CNS disorders, also in this study improvement was most consistently recorded in the area of cognition, adaptative functioning and response to environmental stimuli (Table 3). This finding is in line with oxidative damage being more pronounced in the superior temporal gyrus, frontal cortex and cerebellum, as compared to parietal and occipital cortices (14–18). Interestingly, higher response rates were also recorded in neurodevelopmental disorders characterized by cognitive deficits, i.e., genetic and idiopathic forms of Intellectual Disability, whereas lower response rates were recorded in “pure” ASD and especially in ADHD (see Section Results). Although for the latter the available sample is far too small to allow any firm conclusion, hyperactivity was also the most frequent side effect (Table 4). Similar observations were reported in most (30, 32), though not all previous studies of ASD (31), and seemingly suggest that MST may not exert favorable effects on children who are already prone to impulsivity and problem behaviors. After i.c.v. administration into the rat brain, Co-Q10 and Co-Q9 reach their highest concentrations in noradrenergic areas A5 and locus coeruleus within 15 min (51): a similar distribution after oral ingestion could contribute to the observed “activating” effect of MST, with improved cognition but also, at least in some children, with negative consequences on behavior and sleep. At the opposite end, all five patients with Phelan-McDermid syndrome responded to MST, although they were all rated as “minimally improved” at the CGI-I. Considering that at baseline these five patients were all rated as “markedly ill” or “severely ill” at the CGI-S and that two of them were adults (20 and 37 y.o.), even a modest, yet sizable improvement in a severe genetic disorder is indeed welcome. Phelan-McDermid syndrome in these patients was due to chr. 22q13.33 deletions of different sizes, all encompassing the *SHANK3* gene, which encodes a synaptic scaffolding protein critical to the functioning of excitatory glutamatergic synapses (52). The neocortex of Shank3 ko mice displays increased oxidative stress in the form of increase nitrosylation, of proteins critical to synaptic function, like calcineurin, and of many mitochondrial proteins involved in energy production (53). It will be interesting to assess using patient-derived iPS cells (54) if in human neurons carrying pathogenic 22q13.33 deletions these same phenomena occur and whether Q10 ubiquinol and/or vitamins E and B produce a measureable improvement in synaptic function and/or in axonal transport.

Our study has several major limitations intrinsic to its experimental design, namely a retrospective approach based on the review of medical charts and a somewhat subjective evaluation of their content, applying the CGI-I scale to written reports of direct medical observations and of accounts provided by parents and therapists. Clearly, pre-/post-treatment testing at fixed time intervals with a pre-determined set of tests would have required a prospective open trial design. Per-/post-testing was performed here in a very small number of patients, including the two cases presented in Figures 3A,B, and not systematically. These results must thus be viewed as preliminary, until replicated applying a rigorous randomized control design. Nonetheless, retrospective chart reviews employing the CGI-I do hold a consolidated, robust and valid role in the stepwise analysis of the efficacy of novel psychoactive drug treatments, which typically starts from case reports and then moves up to retrospective chart reviews, prospective open studies, and finally randomized placebo-controlled trials with different designs (10). And indeed, despite its limitations, our report does provide preliminary evidence that MST may represent a safe and viable therapeutic strategy able to partially ameliorate cognitive, adaptive, social and motor functions in children, adolescents and adults with several neurodevelopmental disorders. Regardless of whether isolated, co-morbid or syndromic, ID may represent the most responsive NDD, but a sizable response rate has also been observed also in ASD, whereas ADHD may require caution at this stage.

In conclusion, the present results encourage further investigations of MST efficacy in neurodevelopmental disorders. Randomized controlled trials will now be necessary to confirm the generalizability of these observations, verifying both efficacy, safety, treatment response rates and therapeutic effect size, separately in each major neurodevelopmental disorder.

## Data Availability Statement

The original contributions presented in the study are included in the article/supplementary materials, further inquiries can be directed to the corresponding author/s.

## Ethics Statement

Ethical review and approval was not required for the study on human participants in accordance with the local legislation and institutional requirements. Written informed consent to participate in this study was provided by the participants' legal guardian/next of kin. Written informed consent was obtained from the minor(s)' legal guardian/next of kin for the publication of any potentially identifiable images or data included in this article.

## Author Contributions

FC, AR, LT, and AMP wrote the first draft of the manuscript. AM, LT, AR, RS, and RK retrieved the clinical data. AMP designed and coordinated the study, analyzed the data, and revised the manuscript. All authors contributed to the article and approved the submitted version.

## Conflict of Interest

The authors declare that the research was conducted in the absence of any commercial or financial relationships that could be construed as a potential conflict of interest.

## Publisher's Note

All claims expressed in this article are solely those of the authors and do not necessarily represent those of their affiliated organizations, or those of the publisher, the editors and the reviewers. Any product that may be evaluated in this article, or claim that may be made by its manufacturer, is not guaranteed or endorsed by the publisher.
